# FTO Is Expressed in Neurones throughout the Brain and Its Expression Is Unaltered by Fasting

**DOI:** 10.1371/journal.pone.0027968

**Published:** 2011-11-30

**Authors:** James S. McTaggart, Sheena Lee, Michaela Iberl, Chris Church, Roger D. Cox, Frances M. Ashcroft

**Affiliations:** 1 Department of Physiology, Anatomy and Genetics, OXION Centre for Ion Channel Studies, Henry Wellcome Centre for Gene Function, Oxford, United Kingdom; 2 OXION Centre for Ion Channel Studies, Henry Wellcome Centre for Gene Function, Oxford, United Kingdom; 3 MRC Harwell, Metabolism and Inflammation, Harwell Science and Innovation Campus, Harwell, United Kingdom; University of Bristol, United Kingdom

## Abstract

Single-nucleotide polymorphisms in the first intron of the ubiquitously expressed *FTO* gene are associated with obesity. Although the physiological functions of *FTO* remain unclear, food intake is often altered when *Fto* expression levels are manipulated. Furthermore, deletion of FTO from neurones alone has a similar effect on food intake to deletion of FTO in all tissues. These results indicate that *FTO* expression in the brain is particularly important. Considerable focus has been placed on the dynamic regulation of *Fto* mRNA expression in the hypothalamus after short-term (16–48 hour) fasting, but results have been controversial. There are no studies that quantify FTO protein levels across the brain, and assess its alteration following short-term fasting. Using immunohistochemistry, we found that FTO protein is widely expressed in mouse brain, and present in the majority of neurones. Using quantitative Western blotting and RT-qPCR we show that FTO protein and mRNA levels in the hypothalamus, cerebellum and rostral brain are relatively uniform, and levels in the brain are higher than in skeletal muscles of the lower limbs. Fasting for 18 hours does not alter the expression pattern, or levels, of FTO protein and mRNA. We further show that the majority of POMC neurones, which are critically involved in food intake regulation, also express FTO, but that the percentage of FTO-positive POMC neurones is not altered by fasting. In summary, we find no evidence that *Fto*/FTO expression is regulated by short-term (18-hour) fasting. Thus, it is unlikely that the hunger and increased post-fasting food intake caused by such food deprivation is driven by alterations in *Fto*/FTO expression. The widespread expression of FTO in neurones also suggests that physiological studies of this protein should not be limited to the hypothalamus.

## Introduction

Obesity is a major public health issue. It predisposes to numerous diseases, including heart disease, type 2 diabetes and cancer, and it imposes a significant economic burden on Western societies. By 2030 it is estimated that over 50% of the world population will be overweight or obese [1]. Monogenic causes of obesity result in dramatic phenotypes but are extremely rare. Genes associated with more common, less severe forms of obesity have recently begun to be revealed by genome wide association studies. In this way, single nucleotide polymorphisms (SNPs) in the first intron of the fat mass and obesity-associated (*FTO*) gene were found to be associated with obesity in numerous human populations [2], [3], [4]. Approximately 16% of individuals of European descent are homozygous for the at-risk (A) allele, and have an ∼1.67-fold increased risk of obesity, weighing on average ∼3 kg more than those with the T allele (rs9939609. In most studies that directly measured food intake, individuals possessing at least one copy of the A allele ate more than those with the T allele [5], [6], [7]. These results are in agreement with those from a large questionnaire study [8]. However, in another study where food intake was measured, *FTO* genotype was not associated with total energy intake, but rather with altered food preference; individuals with the A allele consumed a greater proportion of calories from fat [9]. These results suggest that *FTO* may affect body weight by influencing food intake and dietary composition.

Studies in mice generally support this hypothesis. First, enhanced expression of *Fto* leads to hyperphagia, increased fat mass, and a higher body weight [10]. Second, genetic deletion of *Fto*, or a loss-of-function *Fto* mutation, lead to reduced body weight. Paradoxically, mice lacking *Fto* are relatively hyperphagic despite their lower body weight, although this may be a secondary consequence of their growth defect and failure to thrive [11]. Indeed, mice harbouring a loss-of-function mutation in *Fto* show no alteration in feeding behaviour [12]. Taken together, these data demonstrate that alterations in global expression levels or function of *Fto* in mice are positively and reliably correlated with changes in body weight. However, while global overexpression of *Fto* increases food intake, loss of *Fto* function has variable effects on energy consumption.

Changes in *Fto* mRNA expression in specific brain regions may have different effects to changes in global expression. For example, when *Fto* mRNA levels are directly manipulated in the hypothalamic arcuate nucleus of rats by adenoviral infection of *Fto* or by small interfering RNA (siRNA) against *Fto*, food intake is also altered [13]. However, in this study, increased *Fto* expression in the arcuate nucleus was associated with *decreased* food intake and a consequent reduction in body weight. Thus, *Fto* overexpression specifically in the arcuate nucleus has the opposite effect to global overexpression of the protein.

Appetite and food intake are controlled by the brain, specifically by neurones within the hypothalamus although other brain areas also contribute to feeding behaviour [14]. Thus *FTO* is predicted to influence neuronal activity, although whether it does it directly (via changes in neuronal *FTO* expression/activity) or indirectly via hormones or messengers released from *FTO*-regulated peripheral cells is still unclear. In an attempt to distinguish between these two possibilities several studies have focused on the expression of *Fto* in the brain, and searched for differences in the fed and fasted state, primarily in the hypothalamus.

The effects of food intake on *Fto* expression have, however, been controversial. An increase (in rat hypothalamus [15]), decrease (in mouse arcuate nucleus [16]) or no significant change (in mouse hypothalamus [17]) in *Fto* mRNA levels has variously been reported in response to fasting for 40–48 hours. However, fasting mice for >24 hours, may result in pathophysiological responses as it leads to significant weight loss, depletion of hepatic glycogen stores [18], [19], [20] and loss of ∼60% of fat stores [21]. Fasting mice for longer than 48 hours leads to elevation of glucocorticoid levels and increased mortality [22], [23]. Only one study has examined the effects of short-term (16 hr) fasting [24]: this was reported to lead to upregulation of hypothalamic *Fto* mRNA levels.

With one exception [25], where long-term energy restriction was shown to reduce FTO expression in hypothalamus and brainstem, previous studies have focused on *Fto* mRNA levels rather than protein levels and most have been confined to the hypothalamus. However, as high *Fto* mRNA levels have been identified in many brain regions [15], it is important to determine if expression is differentially regulated by fasting in different brain regions. Protein levels are also required as they may not be fully representative of mRNA levels, at least in the short term, if FTO protein turnover is slow.

In this paper, therefore, we investigate changes in the expression of *Fto* mRNA and FTO protein in various brain regions in response to an overnight (18 hr) fast. Similar studies were carried out on two types of skeletal muscles that are largely composed of fast twitch fibres [26], [27]. Our results show that both protein and mRNA levels of *Fto* are higher in brain than in muscle but do not differ between various brain regions or between the individual muscles. Using immunohistochemistry we further show that *Fto* is expressed in the majority of neurones, where it is confined to the nucleus. We found no difference in protein or mRNA levels, in neuronal distribution, or in nuclear localisation of *Fto* after an 18 hour fast.

## Results

### FTO protein is widely expressed across the brain

**Figure 1:**
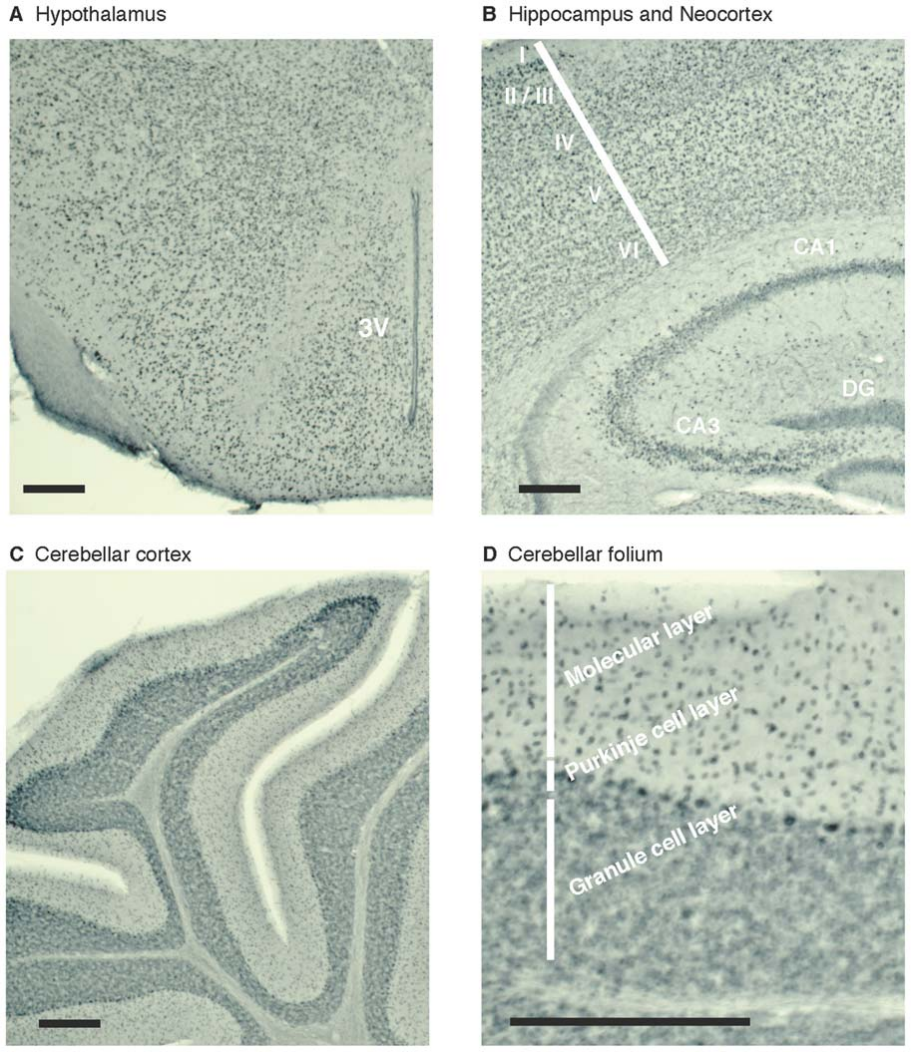
FTO protein is widely expressed in the brain of free-fed mice. Representative images of brain sections from a free-fed mouse (7-week-old female C57BL/6J) probed for FTO protein and stained with DAB (which appears black). (**A**) hypothalamus, (**B**) hippocampus and neocortex, (**C**, **D**) cerebellum. 3V, 3rd ventricle. CA1 and CA3, subfields of hippocampus. DG, dentate gyrus. I–VI, layers of neocortex. Horizontal black bars indicate 200 µm.

Immunohistochemical experiments on brains of free-fed mice revealed that FTO protein is not limited to specific brain regions but is expressed relatively uniformly throughout the brain, including the hypothalamus (Figure 1A), hippocampus and neocortex (Figure 1B), and the cerebellum (Figures 1C and 1D). Brain regions with high neuronal density, such as the granule cell layers of the hippocampus and cerebellum, were especially conspicuous.

**Figure 2:**
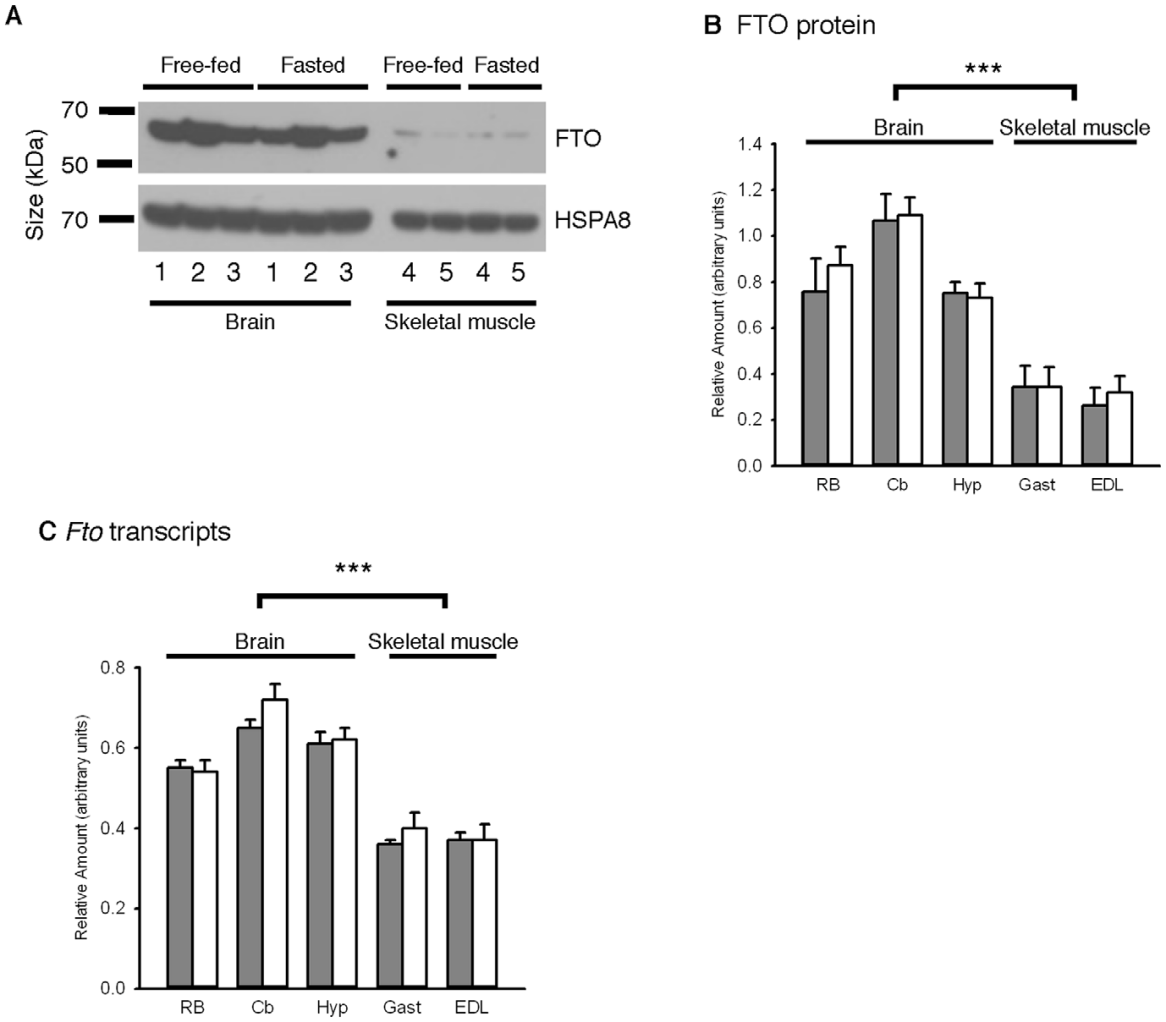
FTO protein and mRNA levels are not altered after ∼18 hours of fasting. (**A**) Representative Western blots of total proteins from free-fed and fasted male C57BL/6J mouse tissues probed for murine FTO and heat shock 70 kDa protein 8 (Hspa8, loading control). Lanes show rostral brain (1), cerebellum (2), hypothalamus (3), gastrocnemius muscle (4) and extensor digitorum longus muscle (5). (**B**) Relative levels of FTO protein, normalised to Hspa8, in tissues from free-fed (grey bars, n = 8) and fasted (white bars, n = 8) 8-week-old C57BL/6J mice. RB, rostral brain; Cb, cerebellum; Hyp, hypothalamus; Gast, gastrocnemius muscle; EDL, Extensor Digitorum Longus muscle. Data are mean±SEM. There were no significant differences between individual brain samples (or between individual muscle samples) based on nutritional status (two-way ANOVA). In free-fed mice, the pooled mean of the three brain samples was significantly higher than the pooled mean of the two muscle samples (one-way ANOVA). ***, p<0.001. (**C**) RT-qPCR quantification of FTO transcript levels in tissues from free-fed (grey bars, n = 8) and fasted (white bars, n = 8) 7-week-old C57BL/6J wild-type mice. RB, rostral brain; Cb, cerebellum; Hyp, hypothalamus; Gast, gastrocnemius muscle; EDL, Extensor Digitorum Longus muscle. Data are mean±SEM. There were no significant differences between individual brain samples (or between individual muscle samples) based on nutritional status (two-way ANOVAs). In free-fed mice, the level of FTO mRNA in each brain region was higher than in either of the two types of muscle (one-way ANOVA with post-hoc pairwise comparisons). ***, p<0.001 for any brain sample versus any muscle sample.

Western blots of total protein isolated from the cerebellum, hypothalamus and rostral brain of free-fed mice confirmed that FTO protein levels were relatively uniform across the brain (Figures 2A and 2B). Although FTO protein levels in the cerebellum were somewhat enriched, there were no significant differences between any of the three brain regions (Figure 2B). Likewise, FTO protein levels in the gastrocnemius and extensor digitorum longus muscles were not significantly different from one another (Figures 2A and 2B). However, there was significantly less FTO protein in skeletal muscle than in brain. Pairwise comparisons between each brain and muscle sample revealed that FTO levels in extensor digitorum longus muscle were significantly lower than those in rostral brain (p<0.05), cerebellum (p<0.001) and hypothalamus (p<0.05). Likewise, FTO levels in gastrocnemius were significantly lower than those in the cerebellum (p<0.001). Although FTO levels in gastrocnemius were not lower than those in the rostral brain and hypothalamus, the differences neared significance (p = 0.06 and p = 0.07, respectively). When protein levels from the three different brain regions of free-fed mice were pooled, and compared to pooled values from skeletal muscle, muscle FTO protein levels were significantly lower than those in brain (p<0.001, Figure 2B).

These results are in excellent agreement with those found for *Fto* mRNA expression using quantitative PCR (Figure 2C). *Fto* mRNA levels in the three brain regions of free-fed animals were not significantly different. Similarly, transcript levels were not significantly different between the two different types of muscle. However, the pairwise comparison between each brain and muscle sample revealed that transcript expression is very significantly less in muscle (p<0.001 in each case).

### FTO is expressed in neurones

To analyse the expression pattern of FTO within specific brain regions in detail, and determine if FTO is confined to neurones, we turned to confocal fluorescence microscopy and double-labelled cells with primary antibodies against FTO ([12]; Figure S1) and NeuN. NeuN is a nuclear protein that labels most neurones, although not cerebellar Purkinje cells [28].

**Figure 3:**
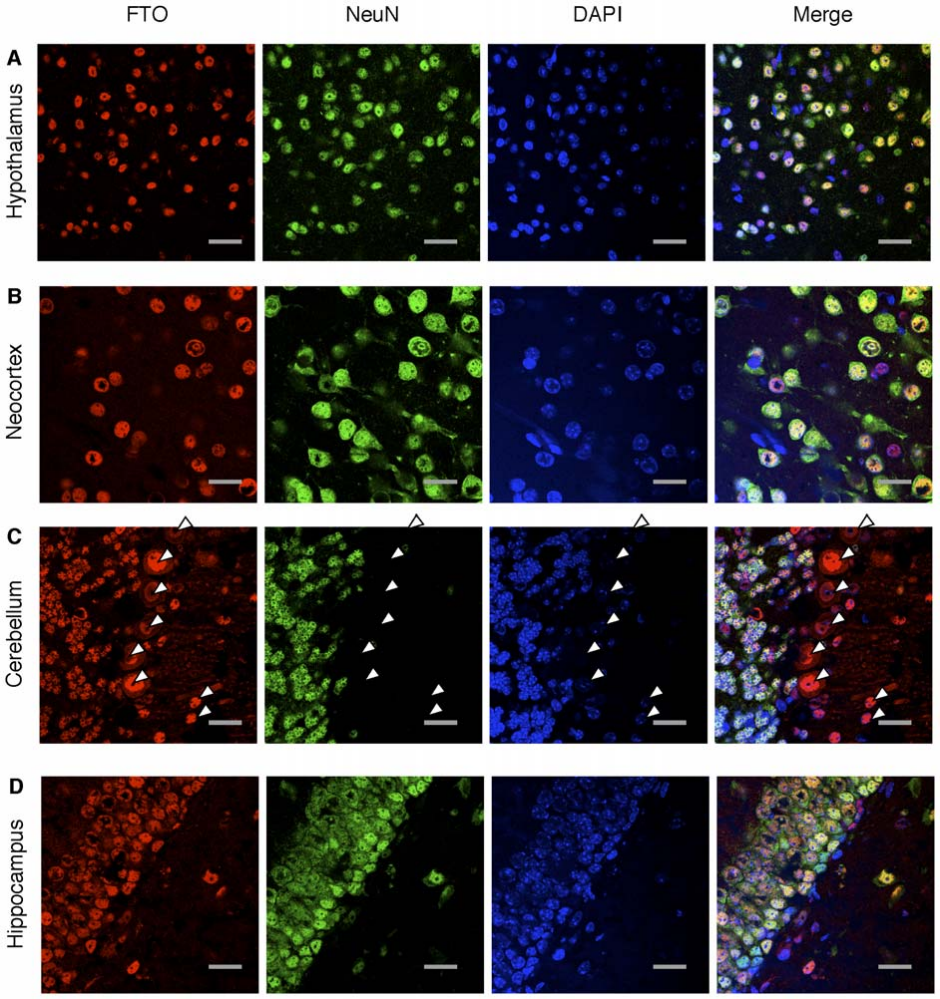
FTO is widely expressed in neurones of the hypothalamus, neocortex, cerebellum and hippocampus. Representative confocal images of sections from the hypothalamus (**A**), neocortex (**B**), cerebellum (**C**) and hippocampus (**D**) of fasted 7-week-old male C57BL6/mice. Sections were probed for FTO protein (red) and Neuronal Nuclear protein (NeuN, green). DAPI was used to stain all nuclei (blue). Note that the FTO antibody labelled cell nuclei but was absent from the nucleolus and cytoplasm. Cells that label for FTO but not NeuN antibodies are indicated by white triangles. Images are representative of 5 mice.

The vast majority (>90%) of cells in the hypothalamus and neocortex that labelled with NeuN were also labelled with FTO (Figures 3A and 3B). This analysis could not be carried out on the images from the cerebellum and hippocampus as the cell density was too high to allow accurate quantification. Nevertheless it is evident that many cells were double labelled (Figures 3C and 3D). Due to the highly organised laminar structure of the cerebellum, it was also possible to identify some cells that were labelled with anti-FTO, but not anti-NeuN (Figure 3C, arrowed). At least some of these cells, such as the Purkinje cells, are neurones that are not labelled with NeuN; nevertheless, it remains possible that some of these cells are not neuronal. FTO-positive but NeuN-negative cells were also seen occasionally in other brain regions, but they were always very few.

Consistent with previous reports [11], [16], [29], FTO expression was restricted to the cell nucleus, being excluded from the cytoplasm and nucleolus (Figure 3).

### Effects of fasting on FTO mRNA and protein expression

Fasting did not significantly change FTO protein (Figure 2B) or mRNA (Figure 2C) levels in any of five tissues that were tested. Interestingly, when *Fto* and reference gene mRNA levels were analysed together in geNorm, *Fto* itself was one of the most stable genes found in both free-fed and fasted animal tissues (File S1). There were no obvious changes in the expression pattern of FTO protein across the brain on fasting (compare Figure 1 with Figure S3). The neuronal localisation of FTO also did not obviously change (compare Figure 3 with Figure S4), with ∼90% of cortical and hypothalamic neurones still expressing FTO. Furthermore, FTO did not alter its subcellular distribution on fasting, and remained confined to the nucleus (Figure S4).

### FTO in POMC neurones

POMC neurones are crucial for regulation of food intake and energy homeostasis [14] and activation of POMC-expressing neurones in the arcuate nucleus of the hypothalamus reduces food intake [30]. We therefore next examined the effect of fasting on FTO protein levels in POMC neurones. We used POMC-Z/EG mice, which express eGFP in POMC-expressing neurones [31], which enabled us to identify POMC neurones by their endogenous GFP fluorescence. We used an anti-FTO antibody and a Texas-red secondary antibody to identify FTO-expressing cells, and DAPI to detect all cells. The number of FTO-positive POMC neurones in the arcuate nucleus of free-fed and fasted mice was counted while blinded to the status of the animals.

**Figure 4:**
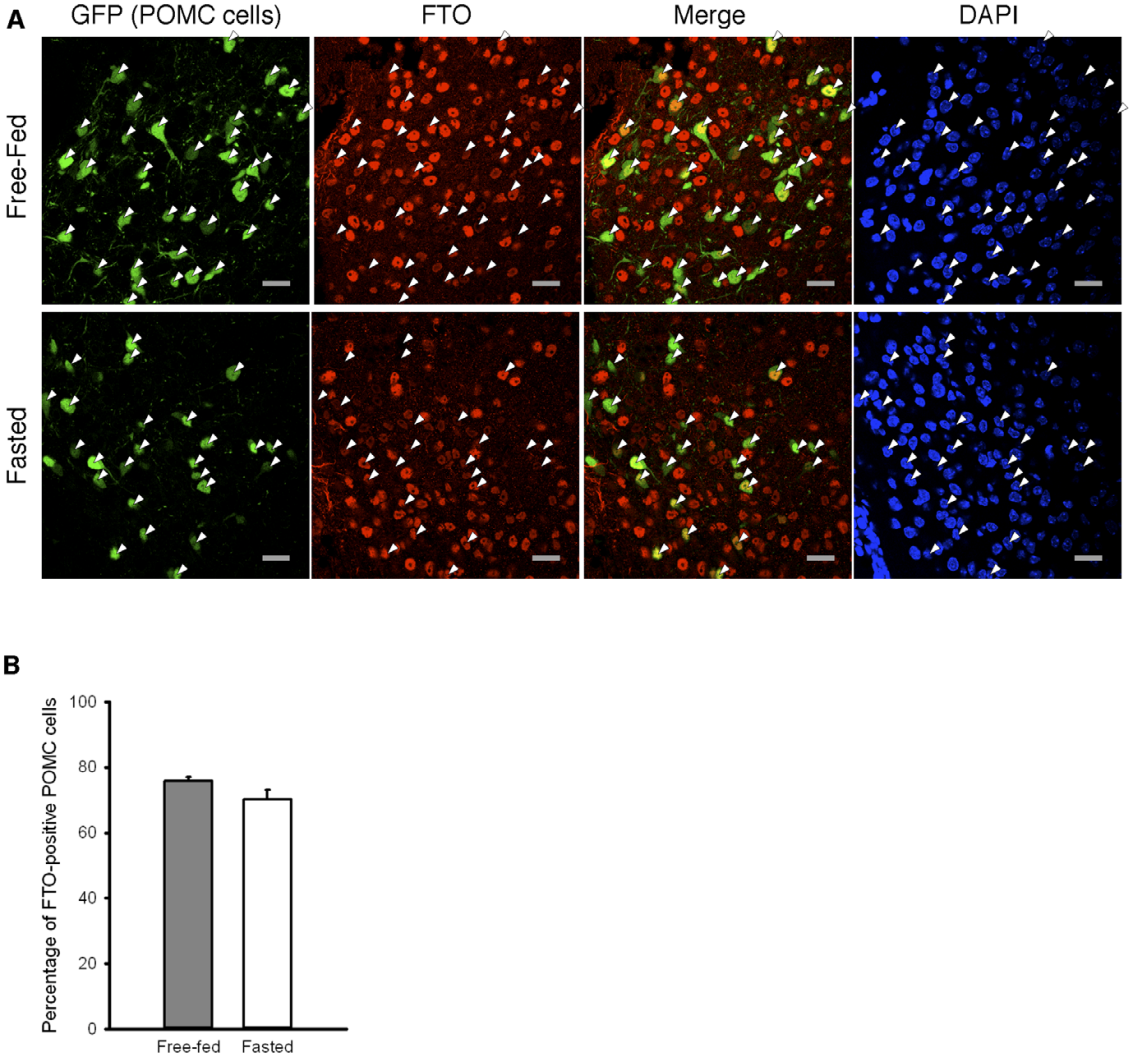
The proportion of POMC cells expressing FTO is not altered by fasting. (**A**) Representative confocal images of hypothalamic brain sections from free-fed (upper panels) and fasted (lower panels) 7-week-old male POMC-Z/EG mice. POMC neurones were identified by GFP expression (green) and are indicated by white triangles. Sections were also probed with anti-FTO antibody (red) and nuclei were stained with DAPI (blue). Images are representative of 5 mice in each case. (**B**) Percentage of POMC cells expressing FTO in free-fed (grey bar, n = 5) and fasted (white bar, n = 5) 7-week-old POMC-Z/EG mice. Data are mean±SEM. There was no significant difference in the percentage of FTO-positive POMC cells based on nutritional status (two-way ANOVA).

Figure 4A shows that FTO is expressed in the nucleus of the majority of POMC neurones. In the free-fed state, approximately 75% of POMC-expressing neurones were labelled with the anti-FTO antibody (Figure 4B). The proportion of FTO-positive POMC cells was not significantly altered after 18 hours of fasting. There were also a significant number of cells that expressed FTO but not GFP, indicating that cells in the arcuate nucleus other than POMC neurones express FTO. This is consistent with a previous report [11].

## Discussion

Our results demonstrate by immunohistochemistry, quantitative Western blot, and Reverse Transcription quantitative PCR experiments that *Fto* is widely expressed, at relatively uniform levels, across the brain. This is in agreement with the widespread distribution of *Fto* mRNA seen in *in situ* hybridisation and RT-qPCR experiments [15], [16], [32].

FTO was found almost exclusively in neurones. In double-labelling immunohistochemistry, more than 90% of NeuN-positive neurones in the neocortex and hypothalamus were also positive for FTO. However, the NeuN antibody does not label all neurones [28], [33], as exemplified by the fact that cerebellar Purkinje cells were clearly not labelled in our experiments. Thus it is possible that an even greater percentage of FTO-positive cells in the neocortex and hypothalamus may be neurones. Interestingly, only ∼75% of GFP-positive (POMC-expressing) neurones in the arcuate nucleus of POMC-Z/EG mice were FTO-positive. This suggests that at least some POMC neurones do not express FTO (we make the assumption that GFP is only expressed in POMC neurones). Thus, it appears that the relative percentage of FTO-expressing neurones varies between 75% and 90%, depending on the brain region.

We observed that both *Fto* mRNA and protein levels were significantly higher in brain than in skeletal muscle. In contrast, previous reports have suggested *Fto* mRNA in skeletal muscle is similar, or greater, than in brain [15], [25]. However, neither of these studies stated which muscles had been analysed, so it is possible that *Fto* levels are higher in skeletal muscles other than gastrocnemius and extensor digitorum longus. However, the physiological relevance of these observations is debatable because *Fto* abundance does not necessarily indicate functional importance.

### FTO expression is not altered by nutritional status

Considerable emphasis in previous studies has been placed on the nutritional regulation of *Fto* expression in the hypothalamus. However, in our experiments there were no significant differences in *Fto* transcript or protein levels either in the hypothalamus or any other brain region in response to an overnight fast.

*Fto* mRNA expression levels in free-fed and acutely fasted rodent hypothalamus have been quantified in several previous studies [15], [16], [17], [24]. It is possible that the significant differences reported for some studies were due to the severity of fasting that was imposed on the animals as pathophysiological processes produced by extended fasting may have resulted in secondary changes in *Fto* expression. The only study that imposed a short period of fasting of similar duration (16 hours) to our study (18 hours) reported that hypothalamic *Fto* mRNA levels were upregulated [24]. There are a few differences between our studies and those of Olszewski and colleagues [24]. They used male C57BL/6J mice whereas we used both male and female C57BL/6J mice: however, we found no differences based on sex. They used 12-week-old animals, whereas we used 7-week-old mice; and they also fed their mice a different chow to ours. Thus, it is possible that dietary and/or age-related factors could account for the difference in our results. Furthermore, as Olszewski and colleagues did not measure protein levels, it is unclear whether FTO function will be altered in their mice.

In summary, our results clearly demonstrate that neither *Fto* mRNA nor FTO protein expression in brain or muscle are affected by overnight fasting. Thus, FTO is unlikely to regulate food intake in response to short-term lack of food via alterations in expression levels in these tissues. Rather, our data suggest that compensatory feeding on reintroduction of food is likely to be mediated (i) via changes in the *activity* of FTO in muscle/brain, or (ii) by changes in gene expression in other tissues, or (iii) it involves an entirely different mechanism. Whether FTO activity is affected by nutritional status is unclear. FTO is an AlkB-like 2-oxoglutarate–dependent nucleic acid demethylase that has been shown to demethylate 3-methylthymine in single-stranded DNA [16] and N6-methyladenosine in nuclear RNA [29]. FTO has also been postulated to serve as a regulator of gene transcription [34]. The activity of many transcription factors is regulated by shuttling them into and out of the nucleus; our data further suggest that FTO does not act in this way to modulate food intake, as its nuclear localisation is unaffected by an 18 hr fast. Finally, while our results do not support the idea that changes in neuronal *Fto* expression contribute to the response to acute starvation, it remains possible that it is involved in the response to small, long-term changes in food supply [25].

## Materials and Methods

### Ethics statement

All experiments were conducted in accordance with the UK Animals (Scientific Procedures) Act (1986) and UK Home Office regulations. The work was performed under certificate of designation number 30/2306 and project licence 30/2668 following approval by the University of Oxford Departments of Physiology, Anatomy & Genetics and Experimental Psychology Joint Departmental Ethics Review Committee.

### Animals

C57BL/6J mice were obtained from an in-house colony. Transgenic POMC-Z/EG mice were congenic on a C57BL/6J background. Mice were housed in same-sex littermate groups of 2–8 and maintained in a temperature and humidity controlled room on a 12 hr light-dark cycle (lights on at 7am), with *ad libitum* access to water at all times. Except when otherwise stated, regular chow food (Special Diets Services RM3) was freely available. Mice (both control and experimental animals) were singly housed for 4 days before fasting, to accustom them to the new environment. All experiments were carried out on 7–8 week old animals. Male and female mice were used. As there were no significant differences based on sex, and no interactions between sex and feeding status, in any of the analyses data from male and female animals are shown pooled in all figures.

### Immunohistochemistry

Mice were terminally anaesthetised by intraperitoneal injection of sodium pentobarbital and perfused intracardially with phosphate-buffered saline (PBS) for ∼2 min followed by 4% paraformaldehyde in 0.1 M phosphate buffer pH 7.4 (PB) for 5 min. The whole brain was removed from the skull and postfixed in 4% paraformaldehyde PB at 4°C for at least 24 hours. Brains were cryoprotected prior to sectioning on a freezing microtome (Leica SM2000R) by immersion in 30% sucrose in PB at 4°C for 18–42 hr. 40–50 µm sections were cut, and rinsed in 0.3% Triton X100 PBS (PBS-Triton) at room temperature

#### DAB-stained immunohistochemistry

Sections were processed according to the ABC method (Vector Labs). They were treated with 10 ml methanol containing 100 µl hydrogen peroxide (30% W/V; Sigma), blocked for 1 hr at room temperature in PBS-Triton with 3% goat serum, and then incubated with FTO antibody ([12]; Figure S1) in PBS-Triton plus 1% goat serum for 24–60 hrs at 4°C. They were then rinsed with PBS, incubated with biotinylated anti-rabbit IgG antibody (Vector Labs BA-1000, 1∶200 dilution) for 1.5 hr at room temperature in PBS-Triton with 3% goat serum, and then treated with the Vectastain Elite ABC kit (Vector Labs, PK6100) for 1–2 hrs at room temperature. Colour was developed with a diaminobenzidine (DAB) kit with metal enhancer (Sigmafast D0426, Sigma). Some sections from each brain were processed identically, but the primary antibody was omitted from the first incubation step. Representative images of these no-primary-antibody control brain sections are shown in Figure S2.

Rinsed sections were mounted on double-subbed slides, left to air-dry overnight, then dehydrated and cleared in ethanol and xylene, and mounted with DePeX (VWR International). Slides were viewed with a Leica DMRB microscope fitted with Zeiss objectives and images captured using a Leica DFC500 camera.

#### Fluorescence immunohistochemistry

Sections were blocked for 1–1.5 hr at room temperature in PBS-Triton containing 5% bovine serum albumin (BSA, Sigma) and 5% goat serum (Sigma). Sections were then incubated with FTO ([12]; Figure S1) and Alexa-488-conjugated NeuN antibodies (Millipore, MAB377X), or with FTO antibody alone (POMC-Z/EG mice) in PBS-Triton with 5% BSA, 5% goat serum for 1 hr at room temperature, and then transferred to 4°C for 18–36 hrs.

For FTO identification, sections were incubated for 1 hr at time at room temperature with biotinylated anti-rabbit IgG (Vector Labs BA-1000, 1∶200 dilution) followed by Texas-Red Avidin D (Vector Labs, A-2006, 1∶100 dilution), then Biotinylated Anti-Avidin D (Vector Labs, BA-0300, 1∶100 dilution) and finally Texas-Red Avidin D again. Sections were rinsed with PBS between each antibody incubation. NeuN fluorescence was increased by probing with an anti-mouse IgG secondary antibody, conjugated to Alexa 488 (Invitrogen A11001). Sections were subsequently incubated for 20 min in PBS with 2 ug/ml DAPI (Sigma, D9542-5MG), to label cell nuclei, washed 3 times in PBS, and mounted on double-subbed slides in Vectashield hardset mounting medium (Vector Labs, H-1400 or H-1500).

Slides were viewed an LSM 510 Meta confocal microscope (Zeiss) and a Plan-Apochromat 63x/1.4NA oil objective (Zeiss). GFP and Alex488 were excited using the 488 nm line of an Argon laser and emitted light was collected between 512 and 565 nm in the Meta channel of the confocal system. Texas Red was excited using the 543 nm line of an HeNe laser and emitted light collected between 597 and 629 nm. DAPI was excited in the two-photon mode with the 740 nm line of a Chameleon laser and emitted light detected between 437 and 480 nm.

For quantification of FTO-positive POMC-expressing cells at least 5 images were acquired for each mouse. The number of cells positive for both FTO (red nuclei) and GFP was expressed as a percentage of the total number of GFP-expressing (POMC) neurones. Quantification was carried out blinded to the feeding status of the mice.

### Western Blotting

Mice were killed by cervical dislocation at ∼10am, and tissues rapidly dissected, snap frozen in liquid nitrogen, ground into a powder and suspended in ice-cold RIPA lysis buffer containing 150 mM NaCl, 1% Nonidet P40, 0.5% Sodium Deoxycholate, 0.1% Sodium Dodecyl Sulphate (SDS), 50 mM Tris, protease inhibitors (G-Biosciences Protecease-50 EDTA free), pH 7.4 with HCl and 10 units of Benzonase (Novagen) to remove nucleotides, for 15 minutes. Non-soluble material was pelleted at 20,000 g for 20 min at 4°C. The supernatant was removed and protein concentrations quantified using the Bio-Rad DC Protein assay. Protein samples were stored at −80°C.

For Western blotting, 35–50 µg of total protein were run on 4–12% Bis-Tris SDS-PAGE gels (NuPAGE Novex, Invitrogen), together with Novex Sharp Pre-stained protein standards (Invitrogen), and transferred to PVDF membranes. Membranes were blocked for approximately 18 hr at 4°C with 5% skimmed milk in 0.1% Tween20 tris-buffered saline (TBST, Sigma) and probed for 1 hr at room temperature with primary antibodies to HSPA8 (Abcam, ab19136) and FTO [12]. HSPA8 was used as the loading control as the *Hspa8* transcript was the most stably expressed reference gene found in RT-qPCR experiments. Blots were then rinsed and probed with horseradish peroxidase conjugated secondary antibody (HRP-anti-rabbit IgG for FTO, GE Healthcare, NA934; HRP-anti-mouse IgG for Hspa8, GE Healthcare, NA931) for 1 hr. Blots were rinsed and bands visualised with enhanced chemiluminescence reagent (SuperSignal West Pico, Pierce) and X-ray film.

Protein density was quantified using the gel analysis function of ImageJ software (National Institutes of Health, USA; [35]) according to the method described here (http://lukemiller.org/index.php/2010/11/analyzing-gels-and-western-blots-with-image-j/). Briefly, HSPA8 protein bands were quantified and converted into relative values, such that the sample with the highest abundance of HSPA8 had a value of 1. The same was done for FTO protein bands. The relative values of FTO were divided by the relative values of HSPA8 to give the relative, normalized quantity of FTO for that sample. The results for each pair of mice were analysed separately.

### RT-qPCR experiments

Free-fed and fasted mice were killed by cervical dislocation at ∼10am, and ‘rostral brain’, cerebellum, hypothalamus, gastrocnemius muscle, and extensor digitorum longus muscle rapidly dissected. ‘Rostral brain’ included all brain regions rostral of Bregma +1.10 mm, corresponding to regions ‘I’ and ‘II’ from Fredriksson et al, 2008 [15]. Samples were immediately immersed in RNAlater solution (Qiagen), and stored at 4°C for 2–3 hours. Tissues were then stored at −80°C until ready to process.

Total RNA was extracted, following the manufacturer's instructions, from 30 mg of thawed tissue using a rotor-stator homogeniser and an RNeasy Mini Kit (brain) or RNeasy fibrous tissue kit (muscle) (Qiagen). All samples were treated with an on-column DNase digestion step to remove traces of genomic DNA. The concentration of purified total RNA was measured in triplicate (and the mean calculated) on a Nanodrop Spectrophotometer (Thermo Scientific) and its quality checked with an Agilent Bioanalyzer. Only samples with an RNA Integrity Number ≥6.5 were processed (most samples were >8).

1 µg of total RNA was reverse transcribed in a 20 µl final volume using the High Capacity complementary DNA (cDNA) Reverse Transcription kit with RNase inhibitor (Applied Biosystems), according to the manufacturer's instructions. A separate microgram of total RNA was processed identically, but without reverse transcriptase (Non-RT control). cDNA samples were then diluted to 4 ng/µl using nuclease-free water (Sigma) and stored at −80°C.

Reverse transcription quantitative PCR (RT-qPCR) was performed using an ABI Prism 7000 Sequence Detection System (Applied Biosystems) using SYBR green reagents (Power SYBR green, Applied Biosystems). All reactions were performed in triplicate using 12.5 µl Power SYBR Green Master Mix, 300 nM primers (forward and reverse), 20 ng (5 µl) of cDNA template and nuclease-free water added to final volume of 25 µl. The reaction cycle comprised an initial denaturation for 10 min at 95°C, followed by 40 cycles of 95°C/15 sec; 60°C/60 sec. Threshold cycle values (Ct values) were measured using the ABI SDS 3000 software (Applied Biosystems).

Primers were designed using Geneious Pro software (Biomatters Inc) and NCBI sequence information (File S2). Primer specificity was checked *in silico* using BLAST and *in vitro* using gel electrophoresis and melt curve analysis. Primer efficiencies were calculated according to the method described in Pfaffl (2001) [36]; all primer pairs had efficiency values of 1.9–2.1, which were used in calculations of relative quantities of transcripts.

cDNA and non-RT control samples were probed for transcript levels of FTO and 6 reference genes (File S2). Each sample was processed in triplicate, for each primer set, and the average Ct value was calculated. The Pfaffl method was used to transform Ct values into relative quantities [36]. The geNorm applet for Microsoft Excel was used to identify the 3 most stable reference genes (*Hspa8*, *Hmbs*, and *Rpl13a*, in order of stability), and the normalisation factor (the geometric mean; Vandesompele et al, 2001). The latter was then used to normalize the relative levels of *Fto*. In a separate analysis, the stability of *Fto* relative to the reference genes was assessed.

For RT-qPCR experiments, Ct values for *Fto* amplification were significantly higher for non-RT controls (30.3±0.4 cycles, n = 80) than for cDNA samples (21.3±0.1 cycles, n = 80; mean ± SEM). The minimum difference between non-RT controls and cDNA samples was 3.1 cycles. Thus, contaminating genomic material had negligible influence on the RT-qPCR results presented in this paper.

### Statistical analyses

ANOVA was used for all statistical analyses. Unless otherwise stated, a two-way ANOVA was carried out in order to assess the impact of both sex and feeding status, and a p value of <0.05 was considered statistically significant. Post-hoc pairwise comparisons were made using the Bonferroni test to clarify the origin of statistically significant results. Separate one-way ANOVAs were used to determine the impact of the five different tissue types on *Fto* mRNA and protein expression in free-fed animals. In addition, a one-way ANOVA was used to compare the mean protein level averaged across all brain regions to that averaged from both muscles.

## Supporting Information

Figure S1 **Specificity of antibody raised against full-length recombinant murine FTO.** (**A**) Representative Western blots. Lanes were loaded with total protein from rostral brain (1), cerebellum (2), hypothalamus (3), gastrocnemius muscle (4), extensor digitorum longus muscle (5) of free-fed (FF) and fasted (Fast) mice. The same blot was probed with anti-FTO (left) and anti-HSPA8 (right) antibodies (the blot was stripped after probing with anti-FTO in order to visualise anti-HSPA8 staining). Both antibodies detected a single band of the appropriate size: FTO was approximately 60 kDa (predicted to be 58 kDa), and HSPA8 was approximately 70 kDa (predicted to be 70 kDa). Blots are representative of those obtained from more than 8 mice. Note that the ‘empty’ lane that separates the brain from the muscle samples contained a pre-stained protein size ladder, which does not appear on the Western blot film. (**B**) Representative Western blots of total protein from brain of wild-type mice (left lane), mice lacking one copy of FTO (middle lane), and mice lacking both copies of FTO (right lane). Above, anti-FTO antibody detected no protein in mice lacking both copies of FTO. Below, beta-actin, which acted as a loading control.
(TIFF)

Figure S2 **DAB staining was specific for FTO antibody.** In the immunohistochemistry experiments, sections from each brain were treated in the same way as experimental sections, but no primary antibody was included in the first incubation step. Representative images of these ‘no-primary-antibody’ control sections from coronal sections of the cerebellum of a control (**A**) and fasted (**B**) brain are shown. Brain sections from the same control (**C**) and fasted (**D**) mice, processed with primary antibody present, are also shown. Note that the control sections are devoid of specific staining. Horizontal black bars indicate 200 µm.
(TIFF)

Figure S3 **The widespread expression of FTO was not altered by fasting.** Sections were probed for FTO protein, which was stained with DAB (black). Representative images of sections from the hypothalamus (**A**), hippocampus and neocortex (**B**), and cerebellum (**C**, **D**) of a fasted 7-week old female C57BL/6J mice. 3V = 3rd ventricle, CA1, CA3 = subfields of hippocampus, DG = dentate gyrus of hippocampus, I–VI = layers of neocortex. Horizontal black bars indicate 200 µm. There were no obvious differences in the pattern of FTO expression in the brain compared with the fed state (Figure 1).
(TIFF)

Figure S4 **The neuronal localisation of FTO was not altered by fasting.** Representative images of sections from the hypothalamus (**A**), neocortex (**B**), cerebellum (**C**) and hippocampus (**D**) of fasted 7-week-old male C57BL/6J mice. Sections were probed for FTO protein (red) and the Neuronal Nuclear protein (NeuN, green). DAPI was used to stain all nuclei (blue). The majority (>90%) of cells in the hypothalamus and neocortex that labelled with NeuN also labelled with FTO. Images are representative of 5 mice. Horizontal grey bars represent 20 µm. There were no obvious differences between images from fasted and free-fed (Figure 3) mice.
(TIFF)

File S1 **Stability of** ***Fto*** **mRNA levels in skeletal muscles of free-fed and fasted mice.** *Fto* and reference gene transcript levels from 8 pairs of free-fed and fasted mice were analysed using geNorm. The stability of the genes is arranged in ascending order (left to right). (**A**) Data for rostral brain, cerebellum and hypothalamus were analysed together and the stability of gene expression is shown. (**B**) Data for gastrocnemius and extensor digitorum longus muscle samples were analysed together and the stability of gene expression is shown. In free-fed and fasted mice, *Fto* itself was one of the most stably expressed genes. In the brain it was at least fourth-most stable in each mouse. In skeletal muscles it was the most stable gene in five of the eight pairs of mice.
(TIFF)

File S2 **RT-qPCR primer information.** Accession number, sequence, amplicon size and efficiency for all primers used in RT-qPCR experiments.
(TIFF)
