# Supplementary figures and images for: FTO Is Expressed in Neurones throughout the Brain and Its Expression Is Unaltered by Fasting
Source: PLoS One. 2011 Nov 30;6(11):e27968. doi: 10.1371/journal.pone.0027968 (PMC3227617; doi:10.1371/journal.pone.0027968)

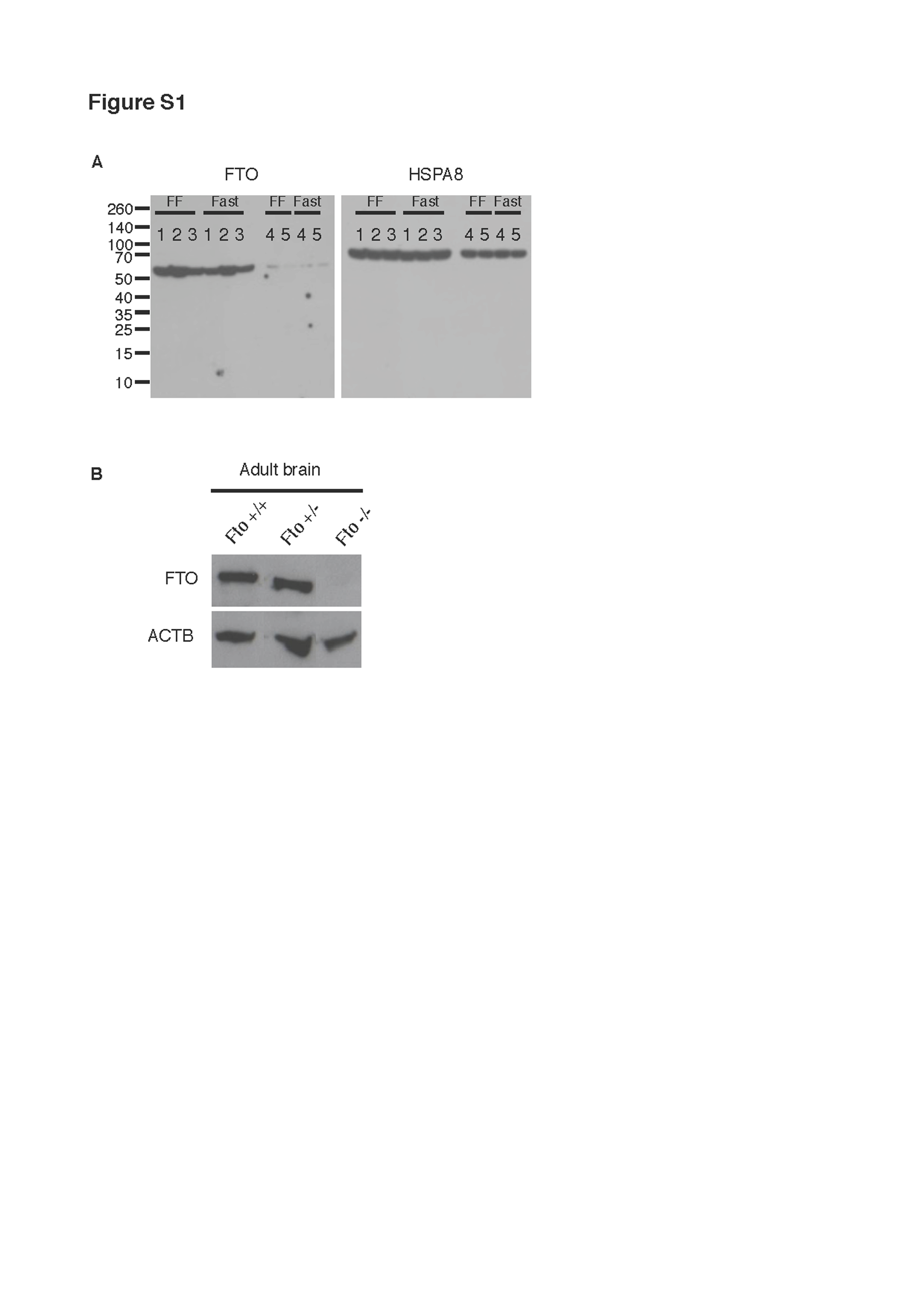

Supplement: Figure S1 — Specificity of antibody raised against full-length recombinant murine FTO. (A) Representative Western blots. Lanes were loaded with total protein from rostral brain (1), cerebellum (2), hypothalamus (3), gastrocnemius muscle (4), extensor digitorum longus muscle (5) of free-fed (FF) and fasted (Fast) mice. The same blot was probed with anti-FTO (left) and anti-HSPA8 (right) antibodies (the blot was stripped after probing with anti-FTO in order to visualise anti-HSPA8 staining). Both antibodies detected a single band of the appropriate size: FTO was approximately 60 kDa (predicted to be 58 kDa), and HSPA8 was approximately 70 kDa (predicted to be 70 kDa). Blots are representative of those obtained from more than 8 mice. Note that the ‘empty’ lane that separates the brain from the muscle samples contained a pre-stained protein size ladder, which does not appear on the Western blot film. (B) Representative Western blots of total protein from brain of wild-type mice (left lane), mice lacking one copy of FTO (middle lane), and mice lacking both copies of FTO (right lane). Above, anti-FTO antibody detected no protein in mice lacking both copies of FTO. Below, beta-actin, which acted as a loading control. (TIFF) [file pone.0027968.s001.tiff]

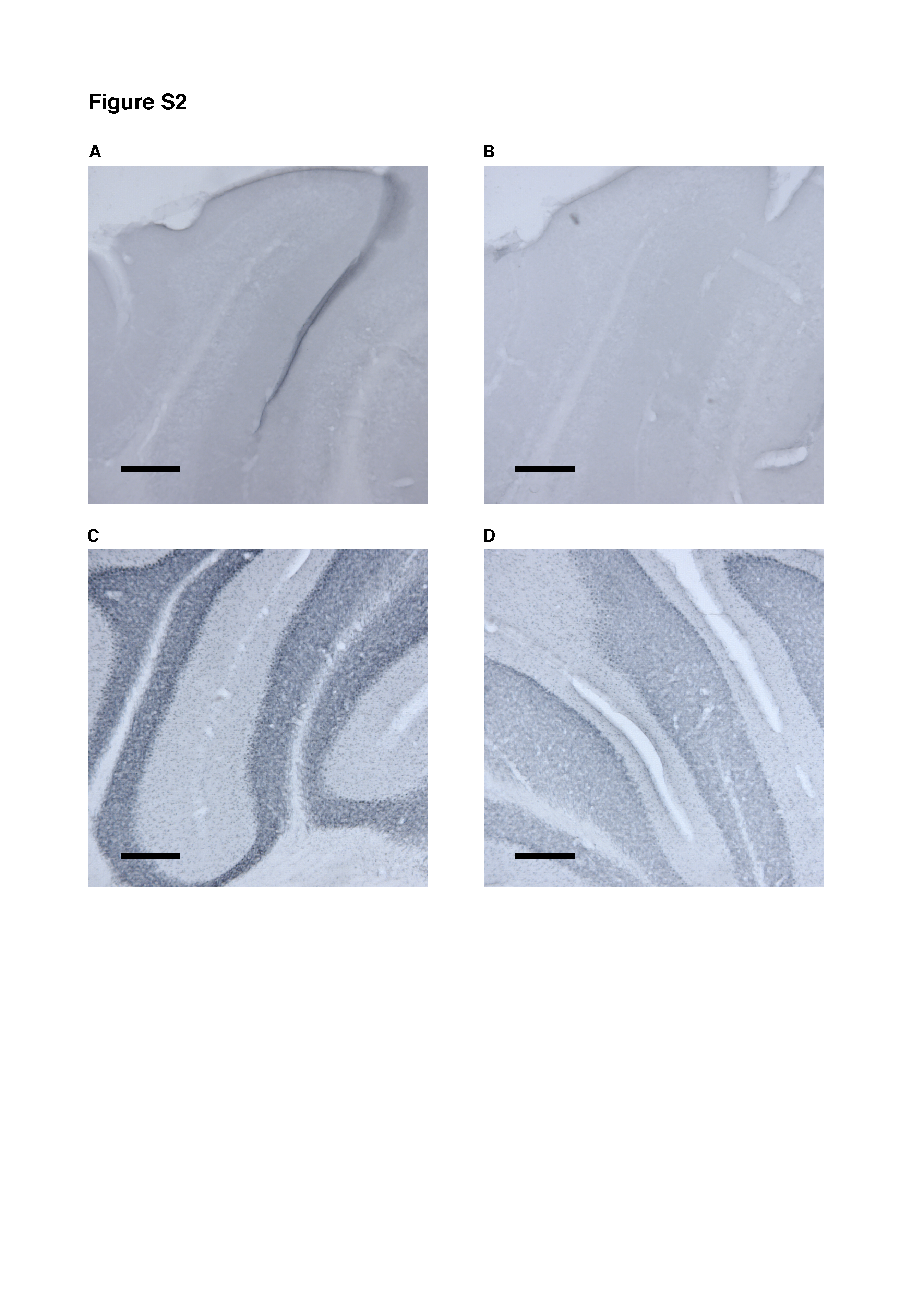

Supplement: Figure S2 — DAB staining was specific for FTO antibody. In the immunohistochemistry experiments, sections from each brain were treated in the same way as experimental sections, but no primary antibody was included in the first incubation step. Representative images of these ‘no-primary-antibody’ control sections from coronal sections of the cerebellum of a control (A) and fasted (B) brain are shown. Brain sections from the same control (C) and fasted (D) mice, processed with primary antibody present, are also shown. Note that the control sections are devoid of specific staining. Horizontal black bars indicate 200 µm. (TIFF) [file pone.0027968.s002.tiff]

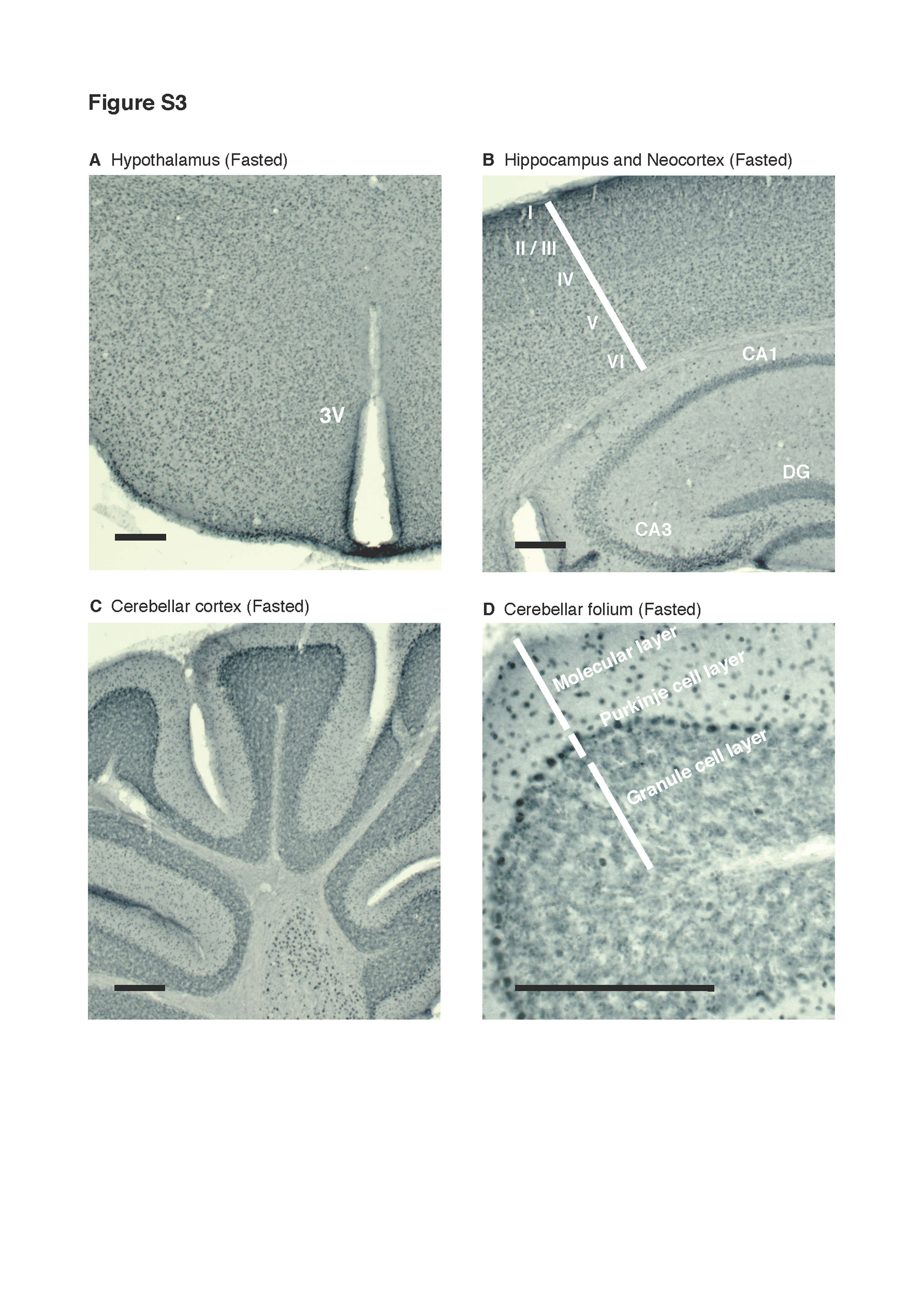

Supplement: Figure S3 — The widespread expression of FTO was not altered by fasting. Sections were probed for FTO protein, which was stained with DAB (black). Representative images of sections from the hypothalamus (A), hippocampus and neocortex (B), and cerebellum (C, D) of a fasted 7-week old female C57BL/6J mice. 3V = 3rd ventricle, CA1, CA3 = subfields of hippocampus, DG = dentate gyrus of hippocampus, I–VI = layers of neocortex. Horizontal black bars indicate 200 µm. There were no obvious differences in the pattern of FTO expression in the brain compared with the fed state (Figure 1). (TIFF) [file pone.0027968.s003.tiff]

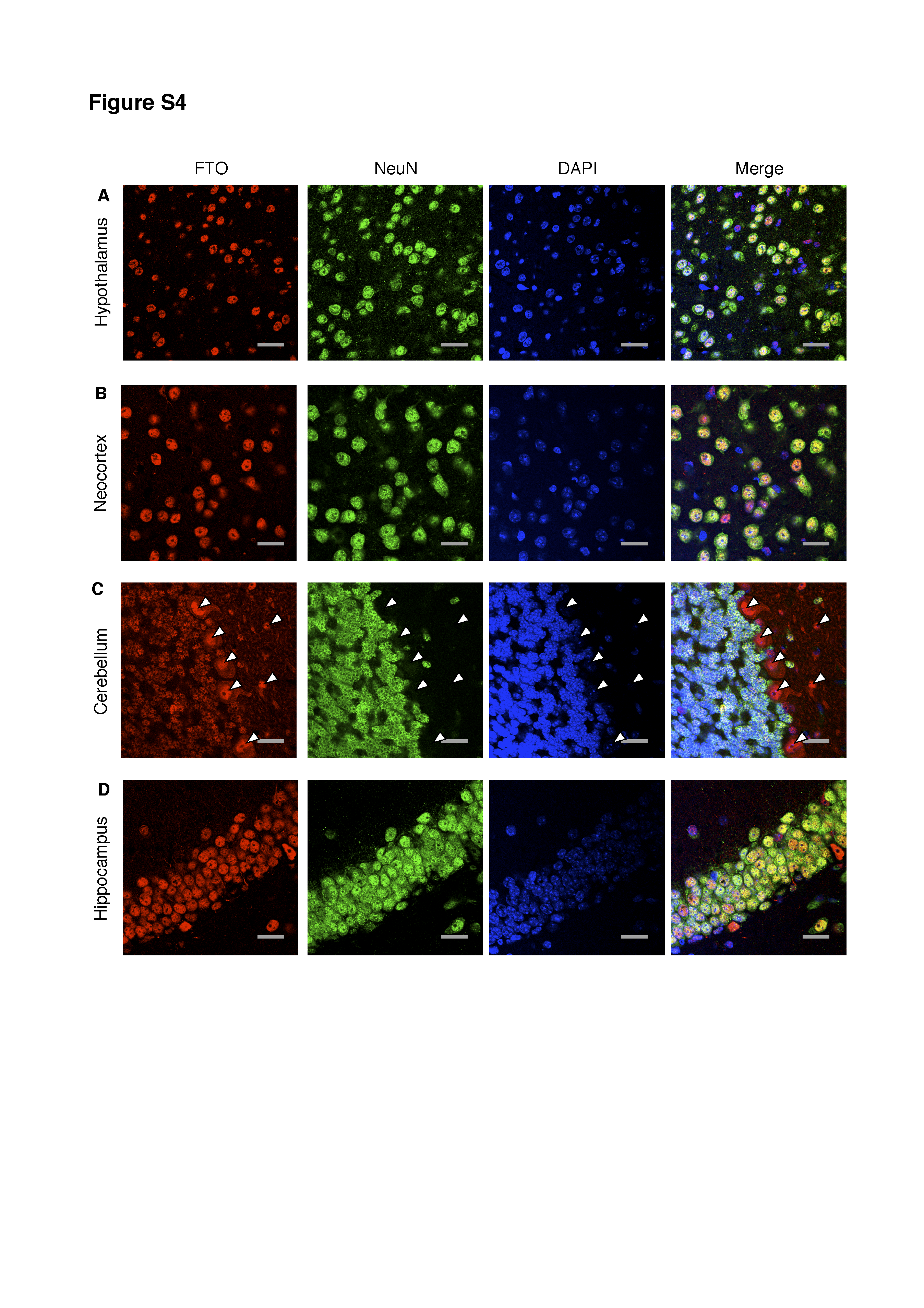

Supplement: Figure S4 — The neuronal localisation of FTO was not altered by fasting. Representative images of sections from the hypothalamus (A), neocortex (B), cerebellum (C) and hippocampus (D) of fasted 7-week-old male C57BL/6J mice. Sections were probed for FTO protein (red) and the Neuronal Nuclear protein (NeuN, green). DAPI was used to stain all nuclei (blue). The majority (>90%) of cells in the hypothalamus and neocortex that labelled with NeuN also labelled with FTO. Images are representative of 5 mice. Horizontal grey bars represent 20 µm. There were no obvious differences between images from fasted and free-fed (Figure 3) mice. (TIFF) [file pone.0027968.s004.tiff]

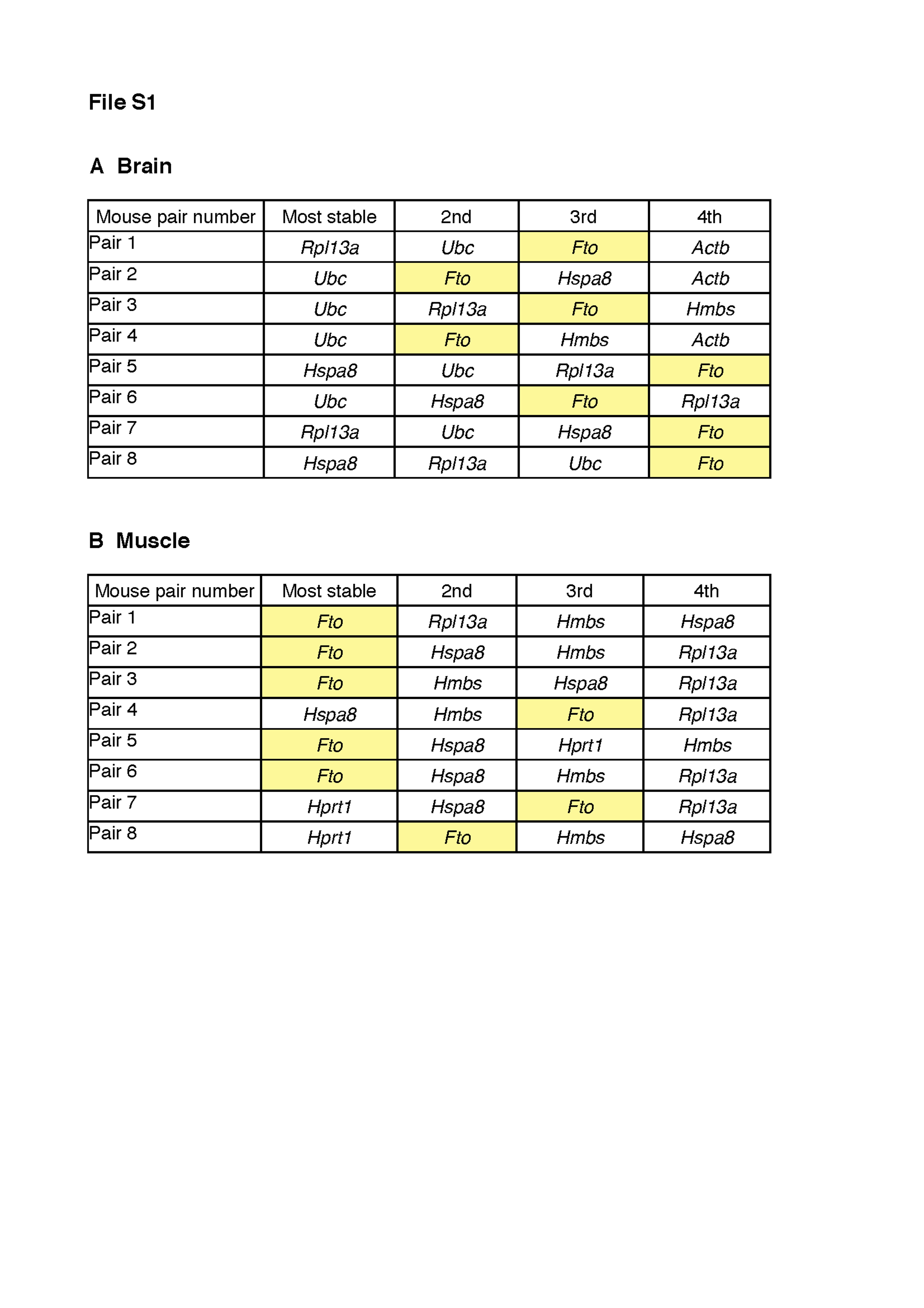

Supplement: File S1 — Stability of Fto mRNA levels in skeletal muscles of free-fed and fasted mice. Fto and reference gene transcript levels from 8 pairs of free-fed and fasted mice were analysed using geNorm. The stability of the genes is arranged in ascending order (left to right). (A) Data for rostral brain, cerebellum and hypothalamus were analysed together and the stability of gene expression is shown. (B) Data for gastrocnemius and extensor digitorum longus muscle samples were analysed together and the stability of gene expression is shown. In free-fed and fasted mice, Fto itself was one of the most stably expressed genes. In the brain it was at least fourth-most stable in each mouse. In skeletal muscles it was the most stable gene in five of the eight pairs of mice. (TIFF) [file pone.0027968.s005.tiff]
